# Randomized study of doxorubicin-based chemotherapy regimens, with and without sildenafil, with analysis of intermediate cardiac markers

**DOI:** 10.1186/s40959-018-0033-2

**Published:** 2018-08-29

**Authors:** Andrew Poklepovic, Yuesheng Qu, Molly Dickinson, Michael C. Kontos, Maciej Kmieciak, Elizabeth Schultz, Dipankar Bandopadhyay, Xiaoyan Deng, Rakesh C. Kukreja

**Affiliations:** 1 Massey Cancer Center and Department of Internal Medicine, Division of Hematology-Oncology, Virginia Commonwealth University, Box 980070, Richmond, VA 23298 USA; 2 Department of Internal Medicine, Virginia Commonwealth University, Box 980070, Richmond, VA 23298 USA; 3 Massey Cancer Center, Virginia Commonwealth University, Box 980037, Richmond, VA 23298 USA; 4 Department of Internal Medicine, Division of Cardiology, Virginia Commonwealth University, Box 980051, Richmond, VA 23298 USA; 5 Department of Biostatistics, Virginia Commonwealth University, Box 980032, Richmond, VA 23298 USA

**Keywords:** Cardioprotection, Chemotherapy, Biomarker, Clinical trial, Ejection fraction, Strain, Anthracycline, Doxorubicin

## Abstract

**Background:**

Doxorubicin chemotherapy is used across a range of adult and pediatric malignancies. Cardiac toxicity is common, and dysfunction develops over time in many patients. Biomarkers used for predicting late cardiac dysfunction following doxorubicin exposure have shown promise. Preclinical studies have demonstrated potential cardioprotective effects of sildenafil.

**Methods:**

We sought to confirm the safety of adding sildenafil to doxorubicin-based chemotherapy and assess N-terminal Pro-Brain Natriuretic Peptide (NT-proBNP) and high sensitivity cardiac troponin I (hsTnI) as early markers of anthracycline-induced cardiotoxicity. We randomized 27 patients (ages 31–77, 92.3% female) receiving doxorubicin chemotherapy using a blocked randomization scheme with randomly permuted block sizes to receive standard chemotherapy alone or with the addition of sildenafil. The study was not blinded. Sildenafil was dosed at 100 mg by mouth daily during therapy; patients took sildenafil three times daily on the day of doxorubicin. Doxorubicin dosing and schedule were dependent on the treatment regimen. Echocardiography was obtained prior to initiation of treatment and routinely thereafter up to 4 years. NT-proBNP and hsTnI were obtained with each cycle before, 1-3 h after, and 24 h after doxorubicin.

**Results:**

Fourteen patients were randomized to receive standard doxorubicin chemotherapy alone (14 treated and analyzed), while 13 patients were randomized to the experimental doxorubicin and sildenafil arm (10 treated and analyzed). No toxicity signal was seen with the addition of sildenafil to doxorubicin-based regimens. There was no statistical difference between the treatment arms in relation to the change of mean left ventricular ejection fraction (LVEF) between the first and last evaluation. In both arms, hsTnI levels increased over time; however, elevated hsTnI was not associated with declines in LVEF.

**Conclusion:**

Adding sildenafil was safe, but did not offer cardioprotection following doxorubicin treatment. Increases in hsTnI levels were observed over time, but elevations during therapy did not correlate with subsequent decreases in LVEF.

**Trial registration:**

This clinical trial (NCT01375699) was registered at www.clinicaltrials.gov on June 17, 2011.

## Introduction

Drugs within the anthracycline class, including doxorubicin, daunorubicin, and idarubicin, are commonly used in chemotherapeutic regimens to treat various solid and hematological malignancies [1–3]. An important anthracycline-related toxicity that impacts cancer survivors is late-onset cardiac dysfunction. Total lifetime anthracycline exposure is positively correlated with congestive heart failure (CHF), thus limiting anthracyclines as a therapeutic option. Dexrazoxane has been evaluated as a cardio-protectant; however, clinical use has been limited as this agent increases hematologic toxicity, and the impact on clinical effectiveness has been controversial. The FDA has approved the use of dexrazoxane to patients with metastatic breast cancer with ≥300 mg/m^2^ of lifetime doxorubicin exposure [4]. Dexrazoxane is also used for extended doxorubicin dosing in patients with metastatic soft tissue sarcoma in combination with olaratumab [5].

The utilization of cardiac markers to predict or assess the cardiac effect of anthracyclines has shown promise. However, no consensus exists for monitoring or predicting patients at highest risk. Baseline elevated levels of high sensitivity troponin T have been shown to be associated with declines in LVEF following anthracycline therapy, however it was not associated with CHF in childhood survivors of cancer [6, 7]. Cardiac troponin I (cTnI) has been shown to identify high-risk patients and predict the development of irreversible heart failure in patients receiving trastuzumab [8]. Elevation of cTnI immediately after treatment and at one month after completion have also been shown to correlate with subsequent declines in LVEF [9]. It has also been reported that natriuretic peptides (eg, N-terminal pro-Brain natriuretic peptide [NT-proBNP]) may serve as a biomarker that can identify later chemotherapy-associated cardiotoxicity [10].

Sildenafil is a phosphodiesterase 5 (PDE5) inhibitor commonly used for pulmonary hypertension and erectile dysfunction. Pre-clinical studies suggested that sildenafil may increase the therapeutic index of doxorubicin through anti-tumor effect and protection from cardiotoxicity [11, 12]. In preclinical murine studies, treatment with sildenafil prior to doxorubicin exposure prevented cardiomyocyte apoptosis and myofibrillar disarray, as evidenced by abnormal desmin distribution, lack of Z-line integrity, and abnormal cytoplasmic desmin aggregation in animals treated with doxorubicin alone [13]. Similarly, long-acting PDE5 inhibitor tadalafil improved LVEF in mice and prevented cardiomyocyte apoptosis following doxorubicin treatment through mechanisms involving up-regulation of cGMP and mitochondrial antioxidant enzyme MnSOD without interfering with the chemotherapeutic benefits of doxorubicin [14].

Based on this preclinical data, we hypothesized that the addition of sildenafil could serve as a cardioprotectant during doxorubicin therapy. The study was not designed to evaluate sildenafil as an adjunct anticancer agent, although other studies are underway in that regard (NCT02466802, NCT01817751). We evaluated the safety of concurrent administration of sildenafil during doxorubicin-based chemotherapy. Additionally, we monitored serum biomarkers before, during, and after doxorubicin exposure for each cycle, to identify the impact, if any, that the timing of marker elevation may have on later systolic dysfunction. Patients were followed up with serial echocardiograms including tissue Doppler imaging to evaluate if changes in LVEF or strain were associated with serum biomarker changes.

## Methods

### Patients

Patients who were undergoing treatment with a chemotherapy regimen with doxorubicin were eligible. Eligibility criteria included: age 18 or older, minimum doxorubicin dose of 40 mg/m^2^, with infusions not more frequently than weekly. In addition, an Eastern Cooperative Oncology Group (ECOG) performance status of 0, 1, or 2 and life-expectancy greater than one year were required. Single agent doxorubicin and combination chemotherapy were allowed. Patients with prior exposure to doxorubicin were included as long as the last dose was greater than 30 days prior to the current doxorubicin-based regimen and their LVEF was greater than 55%. Women of childbearing potential and men agreed to use a medically accepted form of birth control for the duration of study and a minimum of six months after the last dose of doxorubicin. Patients with known CHF, LVEF less than 55%, planned concurrent administration of other investigational agents, swallowing or absorption issues, known hearing loss, hypersensitivity or previous toxicity to study drug, or concurrent chronic nitrate or alpha blocker therapy, were excluded, as were pregnant or nursing women. After six HER2^+^ patients were enrolled, an amendment was written to also exclude patients with planned subsequent therapy with a HER2-directed treatment or other anthracyclines besides doxorubicin. Drugs with strong CYP3A4 inhibitors and/or inducers were not concurrently administered during the study with the exception of short course of aprepitant.

### Treatment

Patients were randomly assigned to receive doxorubicin-based chemotherapy plus sildenafil or doxorubicin-based chemotherapy alone. To ensure recruitment balance between the 2 arms and avoid possible risk for selection bias, a blocked randomization scheme with randomly permuted blocks sizes (unknown to the investigators) was conducted by the biostatistician. Sildenafil was initiated prior to doxorubicin and continued for 2 weeks after the last dose of doxorubicin. Sildenafil dosing was a single 100 mg dose daily and three 100 mg doses on the day of doxorubicin treatment. Each dose of doxorubicin constituted a cycle. The duration of treatment and cumulative dose of doxorubicin were determined by the regimen chosen for treatment and were at the discretion of the treating physician.

### Cardiac function and marker monitoring

LVEF, a surrogate of cardiac function, was monitored with echocardiography. Echocardiography with tissue Doppler and strain imaging was performed prior to treatment; at 3, 6, and 12 months; and then every 12 months for up to 4 years. For the purposes of monitoring patient safety, a clinically significant deterioration in cardiac function was defined as an absolute 10 percentage point decline in LVEF to below 50%, an absolute LVEF of 45% or below, or a 20 percentage point decline in LVEF at any level.

Two-dimensional grayscale harmonic images were obtained in the left lateral decubitus position using an iE33 or EPIQ7 ultrasound system (Philips Medical Systems, Andover, MA) equipped with a transthoracic broadband X5–1 matrix transducer (composed of 3040 elements with 1–5 MHz). 2D grayscale harmonic images were acquired in the 3 standard apical views (2-, 3-, and 4-chamber) obtained at frame rates of ≥50 frames/s. Native 2D images were stored digitally for later off-line analysis.

All echocardiograms were analyzed by a reader blinded to the patient visit number. LVEF was calculated from the apical 4- and 2-chamber views using a modified Simpson biplane method. Myocardial LV deformations were analyzed by speckle tracking using the CMQ software (QLAB 10.3; Philips Medical System, Andover, MA). To assess peak systolic LV longitudinal strain, the endocardial and epicardial borders were traced in the 4C, 3C, and 2C on an end-diastolic frame. The program automatically divided the walls in several segments (LV algorithm based on 17-segment model) and tracked these points on a frame-by-frame basis. When tracking was suboptimal, the borders were readjusted manually.

Pre-treatment levels of cTnI, high-sensitivity troponin I (hsTnI) and NT-proBNP were obtained. High-sensitivity troponin I was determined using a research-phase assay based on LOCI technology and run on a Dimension Vista 1500 System (Siemens Healthcare Diagnostics, Newark, DE). The hsTnI assay has a range of 0.5 to 20,000 pg/mL and a 10% coefficient of variation of 3 pg/mL. NT-proBNP was measured on the Dimension Vista 500 Intelligent Laboratory System (Siemens Healthcare Diagnostics). The limit of detection of the NT-proBNP assay is 0.8 pg/mL.

Blood samples were obtained before every dose of doxorubicin, approximately 1–3 h after each infusion (peak doxorubicin serum concentration), and on day 2 (~ 24 h) following each treatment. These time points were selected to detect both immediate (1–3 h and 24 h after treatment) and delayed (before the next treatment, typically 2–3 weeks later) cardiac injury. Serum was separated and stored at − 80 °C until shipment for batch analysis by Siemens (Newark, DE) for cTnI, hsTnI, and NT-proBNP following the completion of accrual.

### Statistical methods

In addition to the study assignment Arms (doxorubicin/sildenafil; doxorubicin-only), all enrolled subjects were categorized into two Outcome Groups based on an LVEF of less than 50%, which is considered clinically relevant, at their last study visit. Patients were also categorized based on Strain measurement at the 3-year post-treatment echocardiogram (if 3-year strain data was not available then 4-year strain data was used), comparing those with a less than − 17% strain measurement (more negative; better cardiac function) to a greater than or equal to − 17% strain measurement (less negative; worse cardiac function). To assess the longitudinal profiles of hsTnI and NT-proBNP (day 2 sample after each infusion) and their relationship to the explanatory variables such as Arm, LVEF-Group, Strain, Cycle, and their various interactions, separate linear mixed model (LMM) analyses were conducted by using the AR(1) covariance structure. Furthermore, a logistic regression was used to study association of the other explanatory variables to the dichotomous outcome- LVEF-Group, both for hsTnI and NT-proBNP. The 2-sided significance level was set to 5% for assessing significance of the estimated parameters. All analyses were performed using the MIXED or LOGISTIC procedure in the SAS (Statistical Analysis System) software (v.9.3).

Adverse events were characterized using Common Terminology Criteria for Adverse Events (CTCAE) version 4. An early stopping rule for the study was developed based on commonly used doxorubicin regimens [3, 15, 16]. Prior information suggested a cumulative rate of Grade 3 or 4 hematologic and non-hematologic toxicity of 60% or smaller would be acceptable for doxorubicin-based therapies. The stopping rule was designed to ensure that adding sildenafil to doxorubicin did not increase the same type of toxicity to more than 80%. For evaluation of this safety measure, patients underwent clinical and laboratory evaluations. An interim safety analysis examined the difference in LVEF between the two treatment arms using three methods: 1) change in LVEF from the first (baseline) to last visits by the repeated measures analysis of variance (ANOVA); 2) change in LVEF between first and last visits by a pooled t-test; and 3) using levels of LVEF over all visits from baseline up to the 24-month follow-up visit by the repeated measures ANOVA.

## Results

**Fig. 1:**
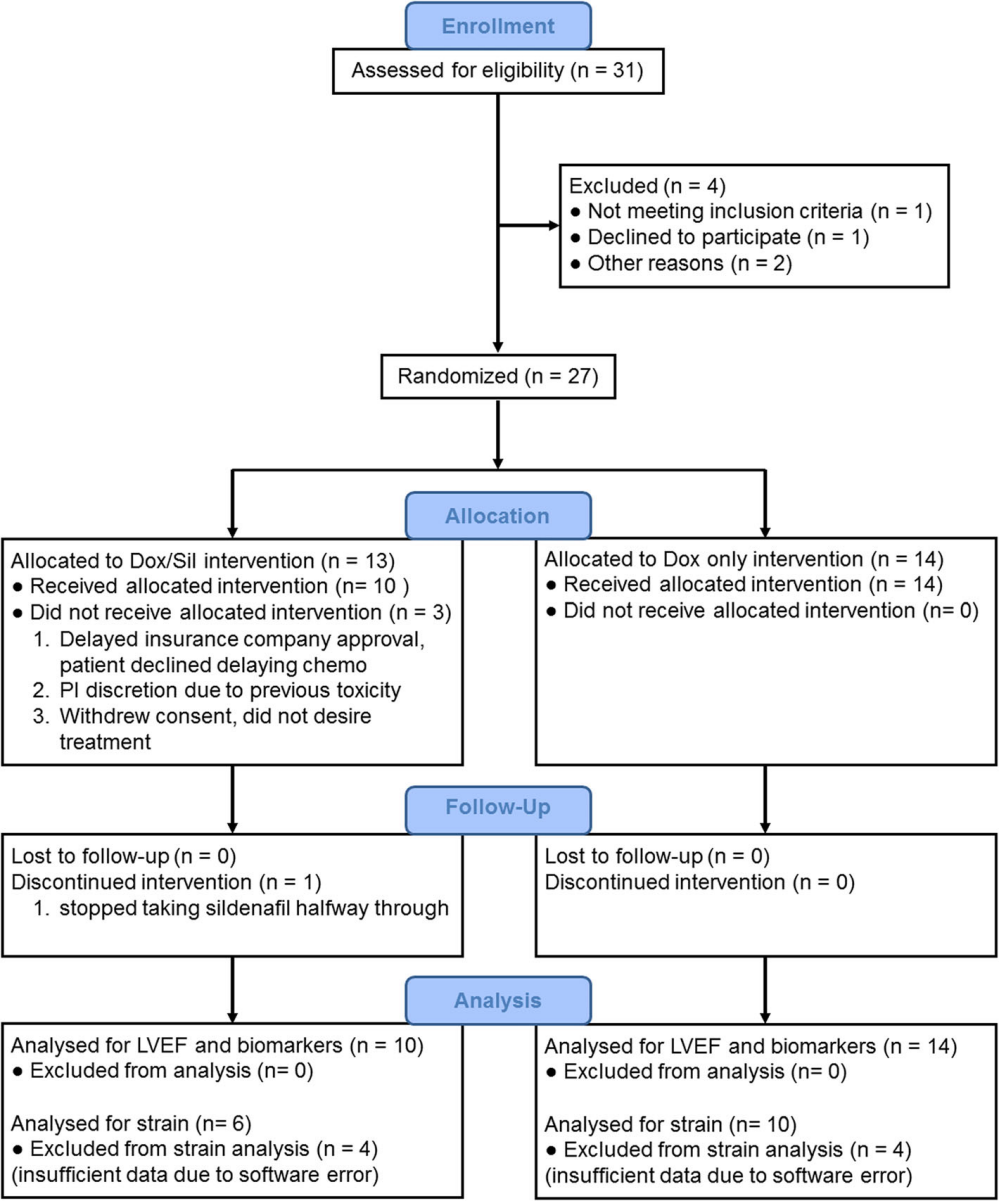
CONSORT diagram. 31 patients were screened, 27 patients were randomized, and 24 patients were treated with doxorubicin alone or a combination of doxorubicin and sildenafil. Patients were followed up for echocardiograms for up to 4 years after completing chemotherapy

Of the 31 patients who were assessed for eligibility, 27 were randomized and 24 ultimately received study treatment, as shown in the CONSORT diagram (Fig. 1). Of these 24 patients, most were female (*n* = 22) (Table 1). Ten patients (median age 57, 40–77) received doxorubicin with sildenafil while 14 (median age 50, 31–67) received doxorubicin only. The most common cancer type was breast cancer (*n* = 20). Five patients had metastatic disease at the onset of treatment. The median total dose of doxorubicin was 240 mg/m^2^ (75–360 mg/m^2^). Smoking risk was more common in the doxorubicin/sildenafil interventional Arm, and cardiac exposure to radiation (defined as left chest wall radiation) was more common in the doxorubicin alone Arm. Two patients in the experimental Arm and 4 patients in the control Arm underwent treatment with trastuzumab following their course of doxorubicin.

**Table 1:** Characteristics of Patients

Characteristic	Doxorubicin-Sildenafil *N* = 10	Doxorubicin-only *N* = 14
	Number (%)	Number (%)
Age		
Median (Range)	57 (40–77)	50 (31–67)
Gender		
Female	9 (90)	13 (93)
Male	1(10)	1 (7)
Race		
American Indian or Alaska Native	0 (0)	1 (7)
Asian	0 (0)	1 (7)
Black or African American	2 (20)	4 (29)
White	8 (80)	8 (57)
Tumor Type		
Breast Cancer	8 (80)	12 (86)
Ovarian Cancer	0 (0)	1 (7)
Sarcoma	2 (20)	1 (7)
Metastatic Disease	2 (20)	3 (21)
Hypertension	3 (30)	4 (29)
Smoking	8 (80)	3 (21)
Diabetes	0 (0)	0 (0)
Radiation to Heart	2 (20)	5 (36)
Trastuzumab	2 (20)	4 (29)

### Safety and tolerability

Sildenafil was generally well tolerated, with well-described sildenafil-associated toxicities (headache, blue-green vision changes, and flushing) reported in a minority of patients (Table 2). Most reported toxicities were Grade 1 or 2. Two patients with Grade 3 headache required dose reductions of sildenafil. There were no Grade 4 toxicities associated with therapy. The threshold for the early stopping rule was not reached, but accrual to the trial was halted for interim safety analysis after a patient who received sildenafil with doxorubicin developed severe LVEF decline. This patient was on adjuvant trastuzumab at that time, and attribution to therapy was unclear. The *p*-value for comparison of LVEF by treatment Arm at the time of the interim analysis was 0.6026. The LVEF from first to last visit was reduced significantly (*p*-value = 0.0009), for both Arms. The time effect did not depend on the treatment Arm (*p*-value = 0.8258). The study was amended and continued to enroll until a second analysis, when it was determined that it was highly unlikely there would be any cardiac protection identified from sildenafil use during the study. It was determined that sildenafil was safe in combination with doxorubicin.

**Table 2:** Adverse Events

CTCAE Term	# Patients (% Patients) Doxorubicin/Sildenafil *N* = 10			
	Grade1	Grade2	Grade3	Grade4
Alanine aminotransferase increased		1 (10)		
Alkaline phosphatase increased	1 (10)			
Alopecia		3 (30)		
Anemia	1 (10)	3 (30)	1 (10)	
Anorexia	2 (20)	1 (10)		
Constipation	2 (20)			
Creatinine increased	1 (10)			
Dry eye	1 (10)			
Dysgeusia	2 (20)			
Dyspepsia	2 (20)	2 (20)		
Edema limbs	1 (10)			
Ejection fraction decreased			3 (30)	
Eye disorders	3 (30)			
Fatigue	3 (30)	3 (30)		
Fever		1 (10)		
Flushing	2 (20)			
Genital edema		1 (10)		
Headache	3 (30)	1 (10)	2 (20)	
Hypocalcemia	2 (20)			
Hypokalemia	2 (20)			
Hypophosphatemia	1 (10)			
Investigations - Other, specify		1 (10)		
Left ventricular systolic dysfunction		1 (10)		
Lip infection		1 (10)		
Lymphocyte count decreased	2 (20)	1 (10)	1 (10)	
Mucositis oral	1 (10)	1 (10)	1 (10)	
Nail discoloration	1 (10)			
Nausea	5 (50)	2 (20)		
Peripheral sensory neuropathy	1 (10)	1 (10)		
Platelet count decreased	4 (40)			
Skin infection	1 (10)			
Vomiting			1 (10)	

### Cardiac analysis

**Fig. 2:**
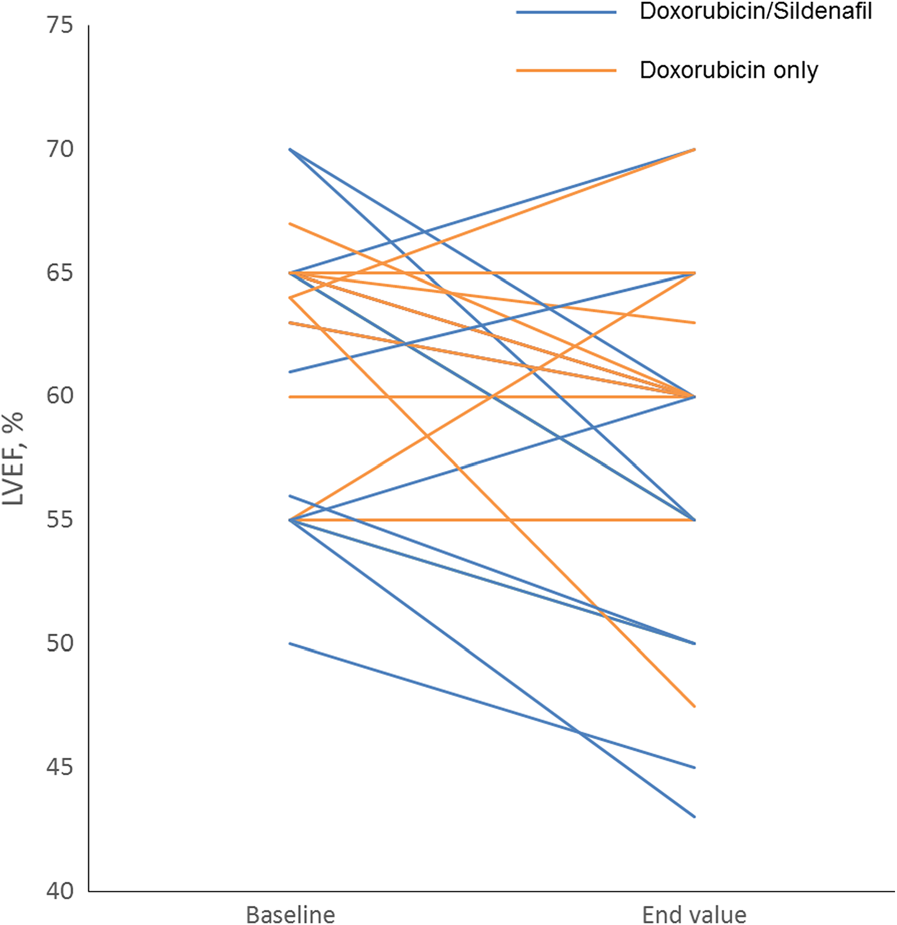
Changes in LVEF. Of the 24 treated patients, 16 experienced LVEF decline following doxorubicin treatment. 7/10 Patients on doxorubicin-sildenafil and 9/14 patients on doxorubicin only experienced LVEF declines of any grade. LVEF change ranged from a decline of 16.5 percentage points to an increase of 10 percentage points with no significant difference between treatment arms (*p* = 0.48)

Left ventricular ejection fraction decreased over time in the majority of patients (Fig. 2). Most patients in both arms experienced declines in LVEF over time (Doxorubicin/Sildenafil *n* = 7/10, Doxorubicin-only *n* = 9/14, total *n* = 16/24). However, the T-test result (*p* = 0.48, Table 3) indicated no significant difference in change of LVEF between the treatment Arms. Both arms, therefore, were pooled in the analysis of cardiac biomarkers in regard to predictive capabilities for cardiac dysfunction.

**Table 3:** Percentage Point LVEF Change by Arm

Arm	Number of Subjects	Mean	Median	Range	*p*-value
Doxorubicin/Sildenafil	10	−4.9	−5.5	20.0	0.4836
Doxorubicin-only	14	−2.9	−3.0	26.5	

For the purposes of identification of patients with cardiac dysfunction resulting from doxorubicin, we defined 2 groups of patients, (LVEF-Group). Patients were allocated into each group for analysis based upon their last follow-up echocardiogram. Cardiac dysfunction patients (*n* = 3) were defined as those having an LVEF of less than 50% at their final study visit. Retained cardiac function patients (*n* = 21) had LVEF greater than or equal to 50%. Patients were also divided into groups by strain outcome. Ten patients had preserved systolic function as assessed by strain measurements less than − 17%, and 6 patients had abnormal systolic function with strain measurements of greater than or equal to − 17%. Eight patients had incomplete data and could not be categorized by strain outcome.

#### Cardiac biomarkers: Troponin I

Serum levels of cTnI were below the lower limit of detection for all patients during the course of the study, and further analysis was not performed.

**Fig. 3:**
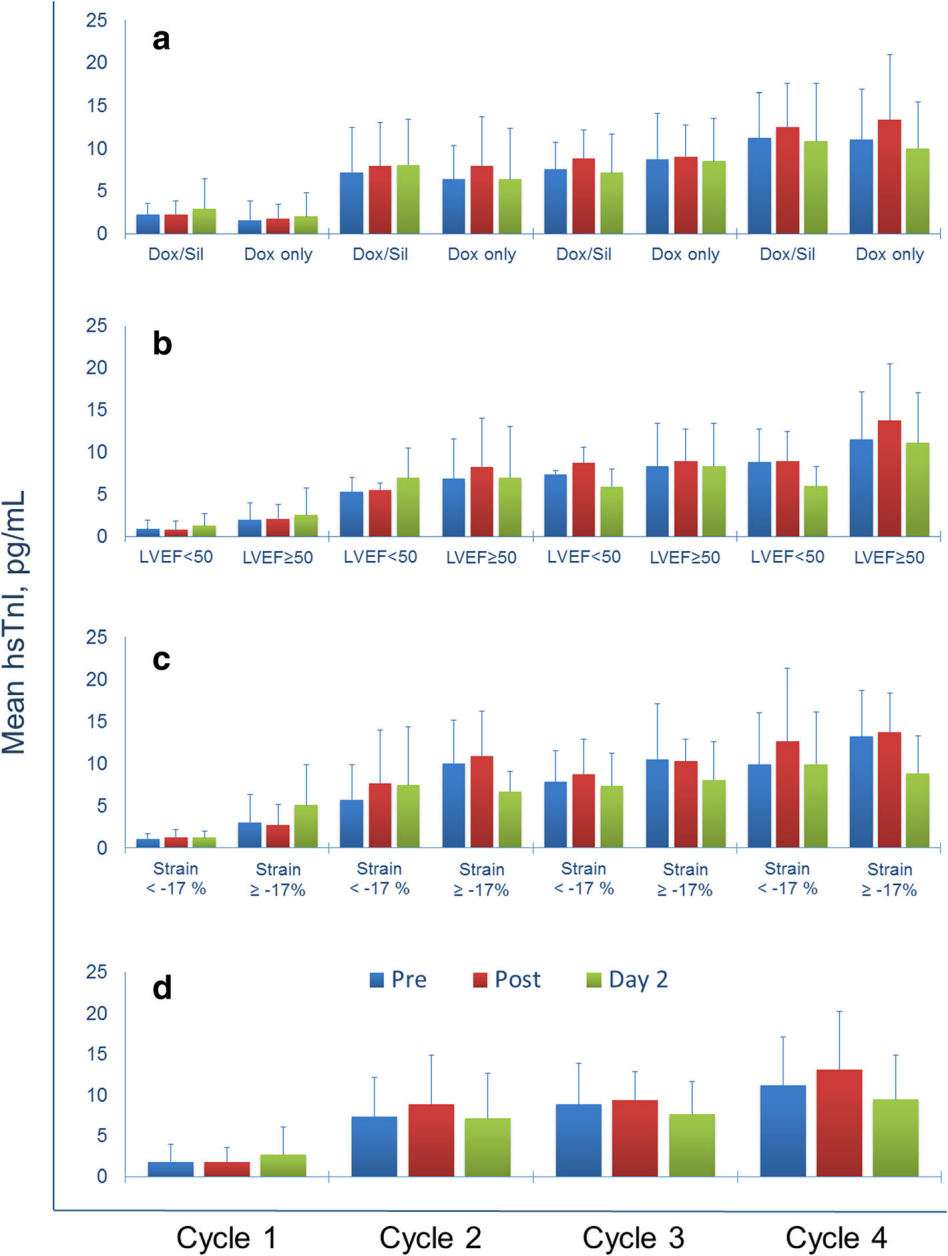
Mean hsTnI by Cycle. **a** Mean hsTnI concentrations increased throughout treatment for all outcome groups, indicating cumulative cardiac injury. All values were below what would be clinically detected in a standard troponin screening. Measuring hsTnI during the period of chemotherapy treatment did not predict later heart function decline as measured by either **b** LVEF or **c** strain. **d** There was a statically significant increase between pre- and post- doxorubicin hsTnI values for patients during cycle 2 and cycle 4 (*p*-value = 0.0029, 0.0059 respectively), an effect not observed with cycle 1 or 3 (*p*-value = 0.7596, 0.2742 respectively)

**Fig. 4:**
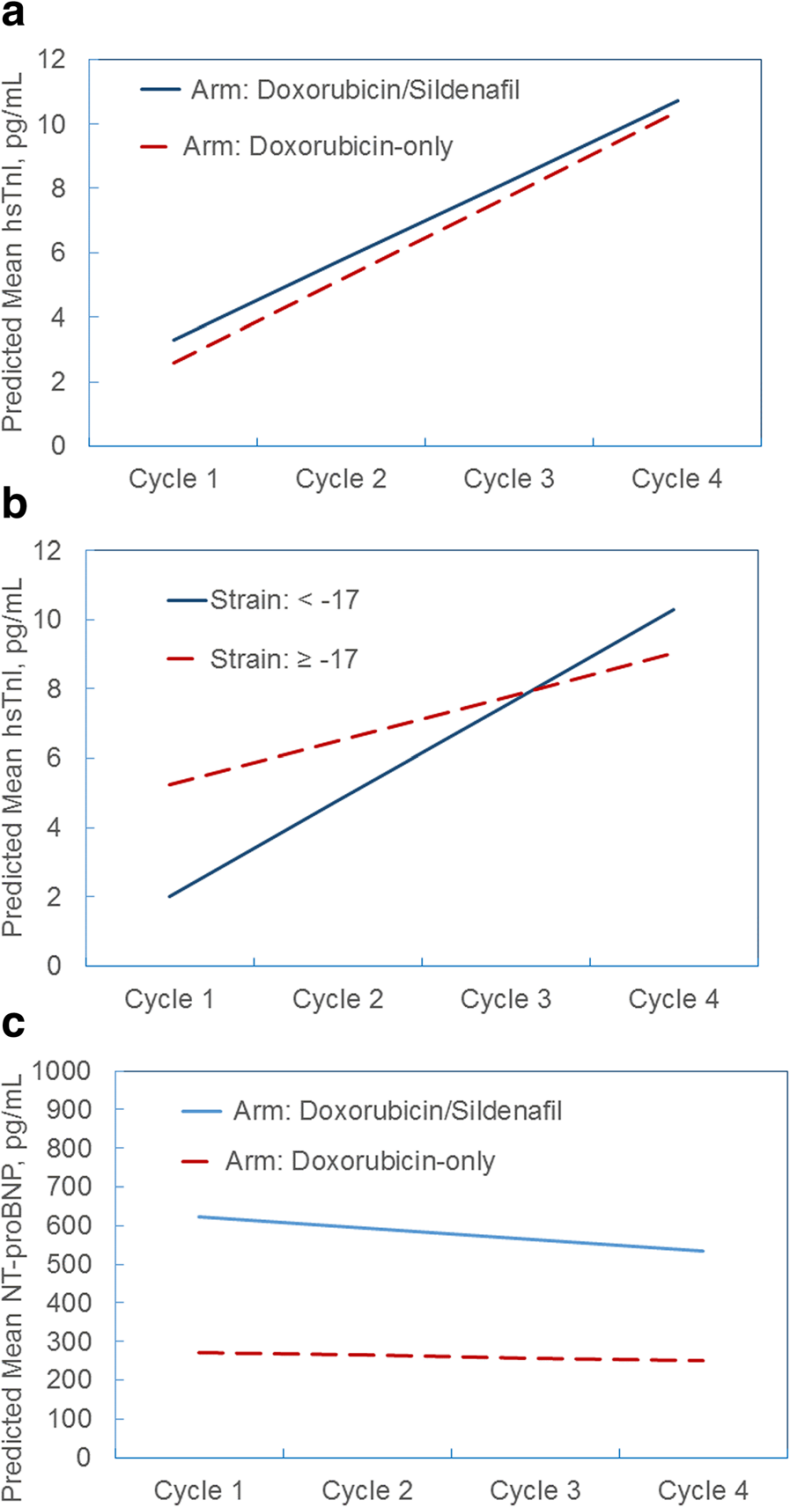
Linear Mixed Model Predictions of Biomarker Concentrations. **a** and **b** Linear mixed model analysis of hsTnI concentrations on the day following doxorubicin infusion demonstrated no significant difference between treatment arms or outcome groups. **c** Linear mixed model analysis of NT-proBNP concentrations on the day following doxorubicin infusion demonstrated significantly higher concentrations in the treatment arm receiving sildenafil

The changes in hsTnI by cycle categorized by Arm and outcome groups (LVEF, strain) are described in Fig. 3 (a-d). The Arm × Cycle interaction was explored using a LMM (Fig. 4a). It was observed that with increasing Cycles, the (mean) hsTnI value increased (estimate = 2.60 pg/mL, *p*-value < 0.0001) for both Arms. No significant difference in (mean) hsTnI value between Arms (doxorubicin-sildenafil; doxorubicin only) was observed (estimate = 0.86 pg/mL, *p*-value = 0.76). Also, the interaction term between Arm and Cycle was not significant (estimate = − 0.13 pg/mL, *p*-value = 0.90). Additionally, no significant difference in mean hsTnI value between the LVEF-Groups (LVEF < 50% vs. LVEF ≥50%) was observed (estimate = 1.30, *p*-value = 0.63, data not shown).

Additionally, to evaluate cardiac injury in the later cycles for the high hsTnI (> 10 pg/mL) group vs the low hsTnI (≤ 10 pg/mL), the SAS procedure GLIMMIX was used to fit a logistic mixed model on this longitudinal data using two explanatory variables, LVEF-Group and Cycle. The estimated coefficient for Cycle is positive (0.6343) and significant at the 5% level (*p*-value = 0.0179), implying progressive injury. The area under the curve for this analysis is 0.3056.

The relationship between the outcome hsTnI and the explanatory factors Strain, Cycle, and Strain × Cycle interaction was explored using a LMM (Fig. 4b). It was observed that none of these 3 factors were significant (*p*-values were 0.12, 0.12, and 0.17, respectively). Strain was not associated with changes in TnI.

#### Cardiac biomarkers: NT-proBNP

**Fig. 5:**
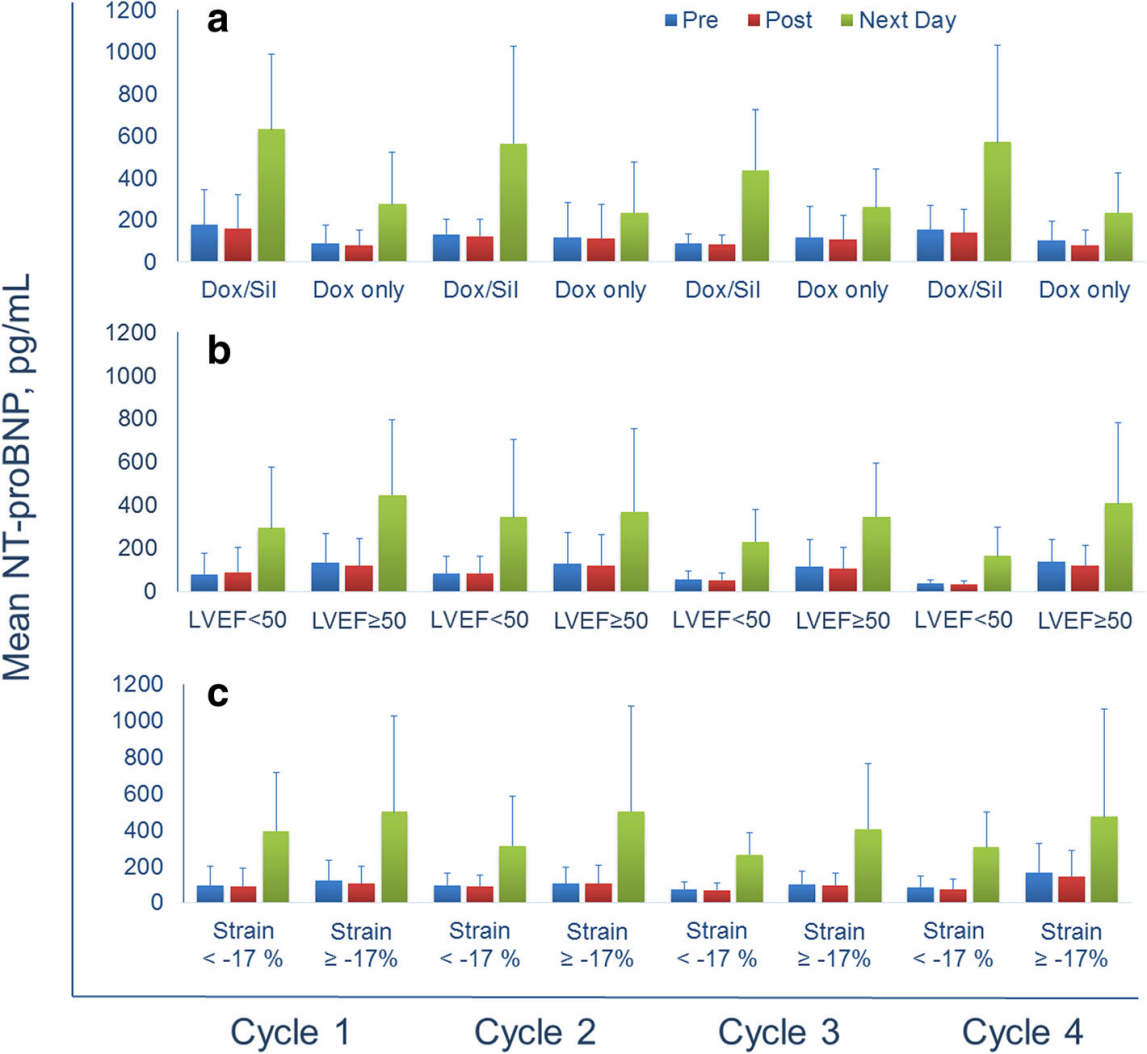
Mean NT-proBNP by Cycle. All **a** treatment arms and **b** and **c** outcome groups experienced temporary spikes in NT-proBNP levels on the day following doxorubicin infusion. However, these levels did not significantly change over the course of chemotherapy

The changes in NT-proBNP by cycle categorized by Arm and outcome groups (LVEF, strain) are described in Fig. 5(a-c). The relationship between NT-proBNP and the explanatory factors Arm, Cycle, and Arm × Cycle interaction was explored using a LMM utilizing day 2 data (Fig. 4c). It was observed that with increasing Cycle, the (mean) NT-proBNP did not change significantly (*p*-value = 0.76). However, a significant difference in (mean) NT-proBNP value between Arms (Doxorubicin/Sildenafil vs. Doxorubicin-only) was observed using day 2 data (estimate = 374.44 pg/mL, *p*-value = 0.034). The interaction term between Arm and Cycle was not significant (estimate = − 22.4 pg/mL, *p*-value = 0.66).

The relationship between the outcome NT-proBNP and the explanatory factors LVEF-Group, Cycle, and LVEF-Group × Cycle interaction was explored using a LMM. It was observed that none of these 3 factors were statistically significant: LVEF-Group (*p*-value = 0.63), Cycle (*p*-value = 0.40), and LVEF-Group × Cycle (*p*-value = 0.64).

To assess interaction between Arm and LVEF-Group for predicting NT-proBNP, the explanatory factors Arm, LVEF-Group, Cycle, and the interaction term Arm × Group were regressed on NT-proBNP using a LMM. We observe that the interaction term Arm × Group was not significant (*p*-value = 0.63); all other factors were also not significant: Arm (*p*-value = 0.073), LVEF-Group (*p*-value = 0.10), and Cycle (*p*-value = 0.48). The relationship between NT-proBNP and the explanatory factors Strain, Cycle, and Strain × Cycle interaction was explored using a LMM. It was observed that none of these 3 factors were significant (*p*-values were 0.74, 0.83, 0.58 respectively).

#### Cardiac biomarkers: Threshold evaluation

As part of the peer review process, the question of identification of patients with highest biomarker values potentially having the greatest risk of subsequent cardiac dysfunction was raised. To address this, threshold levels of hsTnI and NT-proBNP were chosen at 10 pg/mL and 300 pg/mL, respectively. At all 3 measurement time points (pre-treatment, post-treatment, and day 2), the Fisher’s exact tests revealed no significant evidence to support that the subjects with LVEF change greater than 10% were more likely to have high levels of hsTnI or NT-proBNP. The Fisher’s exact tests also showed no significant evidence to support that the subjects with strain ≥ − 17% were more likely to have high levels of hsTnI or NT-proBNP. These analyses are presented in 2 × 2 tables (Tables 4 and 5).

**Table 4:** Threshold Evaluation of hsTnI

		Pre hsTnl		Post hsTnl		Day 2 hsTnl	
		≤10 pg/mL	> 10 pg/mL	≤10 pg/mL	> 10 pg/mL	≤10 pg/mL	> 10 pg/mL
LVEF Change							
N	< 10% (*N* = 17)	10	7	10	7	11	6
Row %		58.82	41.18	58.82	41.18	64.71	35.29
Col %		76.92	63.64	76.92	63.64	73.33	66.67
N	≥ 10% (*N* = 7)	3	4	3	4	4	3
Row %		42.86	57.14	42.86	57.14	57.14	42.86
Col %		23.08	36.36	23.08	36.36	26.67	33.33
	Total	13	11	13	11	15	9
Strain Group							
N	< −17 (*N* = 10)	7	3	6	4	7	3
Row %		70	30	60	40	70	30
Col %		87.5	37.5	85.71	44.44	70	50
N	≥ − 17 (*N* = 6)	1	5	1	5	3	3
Row %		16.67	83.33	16.67	83.33	50	50
Col %		12.5	62.5	14.29	55.56	30	50
	Total	8	8	7	9	10	6

**Table 5:** Threshold Evaluation of NT-proBNP

		Pre NT-proBNP		Post NT-proBNP		Day2 NT-proBNP	
		≤300 pg/mL	> 300 pg/mL	≤300 pg/mL	> 300 pg/mL	≤300 pg/mL	> 300 pg/mL
LVEF Change							
N	< 10% (*N* = 17)	13	4	13	4	6	11
Row %		76.47	23.53	76.47	23.53	35.29	64.71
Col %		68.42	80	65	100	75	68.75
N	≥ 10% (*N* = 7)	6	1	7	0	2	5
Row %		85.71	14.29	100	0	28.57	71.43
Col %		31.58	20	35	0	25	31.25
	Total	19	5	20	4	8	16
Strain Group							
N	< −17 (*N* = 10)	9	1	9	1	5	5
Row %		90	10	90	10	50	50
Col %		69.23	33.33	64.29	50	71.43	55.56
N	≥ − 17 (*N* = 6)	4	2	5	1	2	4
Row %		66.67	33.33	83.33	16.67	33.33	66.67
Col %		30.77	66.67	35.71	50	28.57	44.44
	Total	13	3	14	2	7	9

## Discussion

Anthracyclines are common therapeutic options with curative and palliative intent in many types of cancer. Patients with early stage disease who receive therapy with curative intention can develop delayed systolic cardiac impairment, which diminishes the benefit of anthracyclines and impacts survivorship. Therefore, it is pertinent to develop therapies that can mitigate the cardiotoxicity of treatment and to identify those who are at highest risk of developing subsequent cardiac dysfunction. Dexrazoxane has demonstrated reductions in rates of CHF in patients treated with anthracyclines, and may also be used in selected indications, although use is limited as it increases hematologic toxicity, and the impact on clinical effectiveness has been controversial in some clinical situations. Many cardiologists recommend more intensive management of blood pressure, smoking, diabetes, glucose and cholesterol in patients considered to be a higher risk of late cardiac dysfunction from anthracyclines. Beta blocker and angiotensin receptor blocker therapy can also be employed to help prevent cardiac dysfunction, although their benefit has been inconsistent [17, 18].

Our data demonstrated that sildenafil in combination with doxorubicin-based chemotherapy was safe and generally well tolerated. This study did not demonstrate that sildenafil was cardioprotective, although it was underpowered to detect any modest benefit. Most patients (7/10) in the sildenafil arm had an LVEF decline, further suggesting against cardiac protection, although the change in LVEF over 4 years of follow-up was modest with an approximately − 5% decline. Additional long term followup could prove useful, as some patients develop cardiac toxicity much later than the surveillance time-frame of this study [19].

One patient in the doxorubicin-sildenafil arm experienced symptomatic LVEF decline with severe heart failure. Her LVEF measured 50% at baseline and 69% at the 6-month follow-up visit, and she was treated with trastuzumab following completion of the course of doxorubicin. However, at her 1-year follow-up visit, this patient’s LVEF was 25%, and she was clinically symptomatic. By the time of the 3-year follow-up visit, her LVEF had recovered to 45%. Overall, 5 patients on the study experienced a drop in LVEF to below 50%. Four of these were in the sildenafil arm, and 2 of those 4 received trastuzumab. Two patients experienced recovery from nadir LVEF to a value > 50%.

### Biomarkers

Previous studies have reported that increases in troponin, including hsTnI, occurring after chemotherapy can identify patients who are likely to develop systolic dysfunction [20]. In contrast, in our study, values of hsTnI did not identify patients at risk of clinically significant LVEF declines (< 50%). A unique aspect of our study was the serial assessment of hsTnI pre-treatment, immediately post-treatment, and 24–48 h later. The immediate post-treatment values (during known periods of peak doxorubicin serum concentrations [21]) were increased compared to the pre-treatment values (Fig. 3, panel d), but subsequently decreased over the next 24 h. This is consistent with acute myocardial injury resulting from the doxorubicin. However, serial monitoring (pre-infusion, post-infusion, and day 2) around the day of doxorubicin therapy offered no additional value, as there appeared to be no associated relationship between hsTnI values immediately following doxorubicin administration and subsequent cardiac dysfunction. However, given that the greatest evidence of troponin leak occurred shortly following doxorubicin administration, this may serve as a useful timepoint for future biomarker studies evaluating anthracycline cardiotoxicity. Day 2 hsTnI values did not appear to demonstrate any potential for added utility. In addition, most patients undergoing treatment with doxorubicin in neoadjuvant breast cancer no longer return to the clinic on day 2 after chemotherapy given advances in growth factor support delivery systems, and the results of this study provide no evidence that doing so only for biomarker testing would provide any added prognostic information. In this study hsTnI values increased over time, suggesting subclinical cardiac injury, associated with increased hsTnI release occurring with each doxorubicin dose and progressively higher values with increasing cycle number. Additional studies with more patients and longer follow up could potentially be useful to define whether the increased hsTnI values over time have prognostic value. In larger datasets, tracking new and emerging biomarkers could identify high-risk patients as targets for additional monitoring and intervention.

In our study, NT-proBNP was not a predictive marker. The failure to predict outcomes may be related to the small number of patients who were enrolled, or the lack of significant decline in systolic function. NT-proBNP also failed to identify patients who subsequently developed systolic dysfunction after chemotherapy, a finding reported previously [20]. Elevations in NT-proBNP in the sildenafil arm on day 2 were transient, and thought to be secondary to the vasodilatory effects of high dose sildenafil and were not associated with a long-term negative impact on cardiac function. A similar increase in NT-proBNP with sildenafil treatment was seen in a trial in patients with diastolic heart failure [22].

### Study limitations

This study’s sample size is somewhat small for conducting a full-fledged longitudinal analysis using mixed models. However, to understand the effect of time (measured here in cycles of treatment) and the possible significant differences between the Arms, LVEF-Groups, or Strain groups, a longitudinal analysis was necessary.

Additionally, this study enrolled primarily women with breast cancer, most of whom received less cumulative doxorubicin than doses associated with significant cardiac toxicity (300 mg/m^2^). Therefore, the lack of evidence of a cardioprotective effect cannot be extrapolated to what may be observed in men or patients with other cancer types receiving higher doses of doxorubicin.

## Conclusion

In this pilot study, adding sildenafil to doxorubicin-based chemotherapy did not offer cardiac protection during chemotherapy. Ejection fraction declined over time for most patients (16/24), as expected, following doxorubicin exposure. High sensitivity TnI increased over time, but measuring hsTnI during and immediately after treatment did not identify patients at higher risk for subsequent declines in cardiac function.

## Abbreviations

CHF: Congestive heart failure
CTCAE: Common terminology criteria for adverse events
cTnI: Cardiac troponin I
hsTnI: High-sensitivity cardiac troponin I
LMM: Linear mixed model
LVEF: Left ventricular ejection fraction
NT-proBNP: N-terminal pro-B-type natriuretic peptide
PDE5: Phosphodiesterase 5
